# Development of Recombinant Vaccine against A(H1N1) 2009 Influenza Based on Virus-like Nanoparticles Carrying the Extracellular Domain of M2 Protein

**Published:** 2010-07

**Authors:** R.Y. Kotlyarov, V.V. Kuprianov, A.I. Migunov, L.A. Stepanova, L.M. Tsybalova, O.I. Kiselev, N.V. Ravin, K.G. Skryabin

**Affiliations:** Centre “Bioengineering” Russian Academy of Sciences; Research Institute of Influenza, Russian Academy of Medical Sciences

**Keywords:** influenza, vaccine, M2 protein, nanoparticle, HBc antigen

## Abstract

The conventional vaccines currently being used to deal with influenza are based on a virus obtained in chicken embryos or its components. The high variability of the major immunogenic surface proteins – hemagglutinin and neuraminidase–require the development of strain–specific vaccines that match the antigenic specificity of a newly emerging virus. Recombinant vaccines based on single viral proteins that could be easily produced in standard expression systems are attractive alternatives to traditional influenza vaccines. We constructed recombinant nanosized virus–like particles based on a nuclear antigen of the hepatitis B virus. These particles expose on the surface the extracellular domain of the M2 protein of the highly pathogenic A(H1N1) 2009 influenza virus. The methods of production of these virus–like particles in * Escherichia coli * and their purification were developed. Experiments on animals show that M2sHBc particles are highly immunogenic in mice and provide complete protection against the lethal influenza challenge.

## INTRODUCTION

Influenza is the most common viral disease in humans and animals. Type A influenza viruses vary in their degrees of pathogenicity. In recent years, the H5N1strain has caused local outbreaks of the disease with a high morbidity rate in Southeast Asia. The H1N1 virus originating in swine was behind the flu pandemic that lasted from 2009 to 2010, with an unexpectedly high morbidity rate among middle–aged and high–risk individuals. Given its large amount of phenotypic attributes and its phylogenic origin, the H1N1 virus is akin to the virus that was deemed responsible for the Spanish Flu epidemic that lasted between 1918 and 1920. These characteristics provide evidence of a possible return of a highly pathogenic virus into circulation throughout the human population. The current influenza vaccines are based on a virus obtained from chicken embryos, or from its components [1]. The high variability of the viral surface proteins, hemagglutinin and neuraminidase, leads to the appearance of an epidemic strain every 1–2 years [2], which requires the development of a “standard” strain–specific vaccine at the same rate.

One of the potential causes of antigen variability in the human influenza virus is its recombination (reassertion) with animal flu viruses, which can lead to the appearance of a new, highly pathogenic recombinant virus unfamiliar to the human immune system and, therefore, carrying the risk of a pandemic. At the same time, the development of a traditional vaccine for new strains requires a relatively extended period of time (6 to 9 months), during which the appearance of the new pandemic–causing strain could claim many casualties. As previously stated, the novel pathogenic strain responsible for the 2009 pandemic belongs to the H1N1 class, based on sequences coding of its hemagglutinin and neuraminidase.

Recombinant vaccines are alternatives to traditional methods, and they are based on specific viral proteins. The formulation of these novel vaccines may be achieved in standard producing organisms, such as bacteria or yeast. The use of recombinant vaccines not only eliminates the industry’s dependence on chicken embryos and addresses the general safety concerns associated with vaccines based on the whole pathogen [3], but also creates an opportunity for the development of “universal” vaccines with the use of conservative viral proteins. Moreover, this type of approach allows to produce these vaccines at high speed while “overlapping” the antigen properties of several pandemic viruses.

**Table 1:** Sequence comparison of extracellular domains of M2 proteins of influenza strains of human and animal origins. Amino acids that change relative to the human influenza M2e consensus sequence are underlined.

Host	Strain	М2е peptide sequence
Swine/human	A/California/04/2009	SLLTEVETPTRSEWECRCSDSSD
Human	Consensus sequence	SLLTEVETPIRNEWGCRCNDSSD
Human	A/PR/8/34	SLLTEVETPIRNEWGCRCNGSSD
Avian	A/Chicken/Kurgan/05/2005	SLLTEVETPTRNEWECRCSDSSD
Avian	A/Duck/ Potsdam1402-6/1986	SLLTEVETPTRNGWECKCSDSSD

W. Fiers * et al * . (University of Ghent) analyzed the possibility of developing a universal influenza vaccine based on the extracellular domain of the M2 protein of the influenza virus [4, 5]. M2 is a small transmembrane protein (97 amino acid residues) present in small amounts within the virion, yet it is expressed effectively in the infected cells [6, 7]. An important property of M2 is the conservation of its sequence. The sequence of its extracellular domain (23 amino acid residues), M2e, remains practically unchanged for all type A viruses, which have been extracted from humans since 1933 [4, 8, 9]. However, M2 exhibits a low immunogenicity, and after infection, the immune response against it is practically not activated [10].

The solution to the problem of the low immunogenicity of M2 lies in the protein attaching to the nanosized carrier particle. Such a nanovaccine, when imitating a pathogen, possesses high immunogenicity and is effectively recognized by the human immune system. W. Friers * et al * . used virus–like particles produced by the HBc antigen of the hepatitis B virus as a carrier for the M2e peptide [4, 11]. Mice immunization by M2eHBc particles produced in * E. coli * provided 100% protection against the lethal influenza infection [4]. Besides the HBc, virus–like particles based on the human papilloma virus can be used as carriers of the M2e [12], as well as bacteriophages Q β [13], the papaya mosaic virus [14], and the cowpea mosaic virus [15].

As mentioned above, the sequence of M2e is highly conserved in all human viral strains of Type A influenza; however, in animal strains it differs significantly [16, 17]. The M2e of the swine flu virus A/California/04/2009(H1N1), which was responsible for the 2009 pandemic, differs from the M2e of the human strain in 4 out of 23 amino acid residues (Tabl e 1 ). Such differences may determine the specificity of vaccines based on M2e. In this work, we constructed recombinant particles (M2sHBc–particles) which carry the M2e viral peptide of the swine flu A/California/04/2009(H1N1) and showed that immunization with such nanoparticles provides full protection of vaccinated mice against the lethal challenge by swine flu virus A/California/04/2009(H1N1). At the same time, protection against the avian flu infection, strain A/Duck/Potsdam1402–6/1986 or human strain A/PR/8/34, proved only partial, which emphasizes the necessity of taking into account the sequence differences of M2e of influenza strains of different origins when developing universal influenza vaccines.

## EXPERIMENTAL

**Construction of expression vector pQE–M2sHBc and** *E. coli* **producer strain**. The gene that codes for the hybrid protein M2sHBc was synthesized using a three–step PCR. During the first step, the portion of the M2HBc sequence was obtained as a result of PCR with the primers M2F3 (C GAA TGG GAA TGC CGT TGC AGC GAT AGC AGC GAT GAC CCT) and HBC–R2 (A GGA TCC TCA GCA AAC AAC AGT AGT CTC CGG AAG) and DNA copy of the hepatitis B virus genome as a template. During the second step, the obtained fragment was used as a template for the PCR with the primers M2sF1 (GAA ACC CCG ACC CGT AGC GAA TGG GAA TGC CGT TGC AGC) and HBC–R2. During the third step, a full–sized gene, M2sHBc, was obtained as a result of the PCR amplification with the primers M2sF2 (CTC ATC AGC CTG CTG ACC GAA GTG GAA ACC CCG ACC CGT AGC) and HBC–R2. The fragment obtained, 525 bp, was digested with the restriction enzymes PagI and BamHI, whose recognition sites were entered into the sequence of primers M2sF2 and HBC–R2, respectively, and cloned into the expression vector pQE60 (Qiagen) using sites for NcoI and BamHI. The expression vector pQE–M2sHBc was used in further work. Sequencing verified the absence of the PCR–specified mutations in the synthesized gene.

To obtain the producing strain of M2sHBc, plasmid pQE–M2sHBc was introduced into the * E. coli * strain DLT1270 by transformation. Strain DLT1270, a derivative of the DH10B [18], contained the repressor gene for the lactose operon * lacI* integrated into a chromosome.

**Isolation and purification of the M2sHBc–particles**. Strain DLT1279/pQE–M2sHBc was grown in LB–broth until the midpoint of the logarithmic growth phase (OD_600_ = 0.5) at 37 ^0^C, then IPTG was added to 1mM, and the culture was left to continue to grow for 16 hours at 30 ^0^C. Cells from the producing strain were collected by centrifugation at 3,000 rpm for 30 minutes and were re–suspended in a 50 mM Tris–HCl buffer pH 8.0, which contained 0.5M NaCl, 15mM EDTA, and 20% sucrose, calculating 1ml of buffer per 50ml of culture. Cell suspension was treated with lysozyme (1mg/ml) for 15 min at 4 °C; afterwards, the cells were lysed by sonication. Polyethylene glycol (50% weight/volume) was added to the lysate solution (1/20 volume) and incubated for 30 min at 4° C. Next, it was centrifuged for 20 minutes at 13,000 rpm. A fifth of the volume of the concentrated solution of ammonium sulfate was added to the supernatant, mixed and left to stand for 30 min at 4 °C. The produced protein precipitate was suspended in 1ml of the same buffer and precipitated with ammonium sulfate a second time under the same conditions. The produced precipitate was dissolved in a 1ml 50mM Tris–HCl buffer with pH 8.0, which contained 0.5 M NaCl, 15mM EDTA, and 20% sucrose. The obtained preparation of M2sHBc particles contained, according to the SDS–PAGE, about 90% of the M2sHBc protein with a concentration of ~ 0.5 mg/ml.

**Table 2:** Evaluation of immunogenicity and protectivity of the candidate vaccine based on the M2e peptide of the swine influenza virus.

Group of mice	Number of mice	First immunization	Second immunization	Third immunization	Influenza virus challenge		
A/Duck/Potsdam/1402-6/1986 (Н5N2)	A/California/04/2009 (H1N1)	A/PR/8/34
Experimental (М2sНВс)	60	60 mice with TiterMax Gold Adjuvant 50 μg/mice s.c.^ (1) ^	60 mice with Freund's incomplete adjuvant 50 μg/mice s.c.	60 mice with Freund's incomplete adjuvant 50 μg/mice s.c.	20 mice 5 LD/50	20 mice 5 LD/50	10 mice 5 LD/50
Control	40	PBS	PBS	PBS	15 mice 5 LD/50	15 mice 5 LD/50	10 mice 5 LD/50

subcutaneous injection

**Mice Immunization**. To study the immunogenicity and the protectivity of the candidate vaccine, the immunization scheme was applied with the use of the TiterMax Gold Adjuvant (Sigma) at first immunization and the incomplete Freund’s adjuvant (Sigma) at the following immunizations. Second immunization was conducted three weeks after the first, and the third was done the following week. The immunization outline is shown in Table 2.

Sera were collected 2 weeks after the third immunization, and the antibody titers were determined in the pooled sera of mice of each group (3–5 mice). As negative control, the serum of nonimmunized mice was used. As positive control, monoclonal antibodies to the M2e peptide strain A/Duck/Potsdam/1402–6/1986 (H5N2) were used: they were provided by P.G. Sveshnikov (Russian Research Center of Molecular Diagnostics and Therapy).

**Synthetic Peptides**. As a standard for the determination of M2e antibody synthetic peptides G–11–1 (SLLTEVETPTRNEWECRCSDSSD, corresponding to M2e of strain A/Chicken/Kurgan/05/2005), G19 (SLLTEVETPTRNGWECKCSDSSD, corresponding to the M2e of strain A/Duck/Potsdam1402–6/1986), G26 (SLLTEVETPTRSEWECRCSDSSD, corresponding to the M2e of strain A/California/04/2009), and G18 (SLLTEVETPIRNEWGCRCNDSSD, corresponding to M2e of strain A/PR/8/34) were used.

**ELISA for the titer determination of specific antibodies**. For ELISA, 96–well plates with a high sorption capacity (Greiner, Germany) were covered with synthetic peptides G–11–1, G19, G26, and G18 with a concentration of 5 mg/ml (in the carbonate buffer, pH 9.5–9.6) and kept overnight at 4 °C. Plates were treated with a blocking buffer (0.01 M PBS pH 7–7.4) with 5% FCS for 1 hour at room temperature and washed 3 times with PBS–Tween. The pooled mice sera from each group were analyzed in duplicates. 100 µl of 2–time serum dilutions were added to the well plates (starting with 1:400) in the blocking buffer then incubated for 1 hour at room temperature. As a conjugate, rabbit polyclonal anti–mice IgG (Abcam, Great Britain) were used in a 1:8,000 dilution, marked with a horseradish peroxidase. ТМ B was used as a substrate. The reaction was monitored by UV–Vis spectroscopy at 450 nm. The last dilution of the serum, which had an optical absorption at least twice higher than that of nonimmunized mice, was taken as an antibodies titer.

**Viruses and mice infection** For the infection of the animals immunized by the candidate vaccines, the following influenza viruses, adapted to the mice, were used: A/Duck/Potsdam/1402–6/1986(H5N2), A/California/04/2009 (H1N1), and A/PR/8/34 (H1N1). The virus was administered intranasally in a total volume of 50 μl containing 5LD_50_ to mice anesthetized by ether. The animals were observed daily after infection. The protective properties of the candidate vaccine were evaluated based on two parameters: determination of body weight dynamics and mice survival after infection.

## RESULTS AND DISCUSSION

**Design and production of M2sHBc nanoparticles **. The Hepatitis B nuclear antigen is one of the most effective carriers of antigen determinants. Monomers of this protein, consisting of 183 amino acid residues, self–assemble into icosahedral particles with a 34 nm diameter, made of 240 subparticles organized in dimeric blocks [19]. Two HBc antigen regions can be used for the presentation of foreign peptides on the surface of the HBc particles – the protein N–terminus and the immunodominant loop located between the 75^th^ and 85^th^ amino acid residues of the protein [20–22]. Based on our experience, the introduction of the foreign sequence into the immunodominant loop results, in most cases, in the disturbance of the assembly and/or the solubility of the particles. Therefore, as a site for the introduction of the M2e peptide for the construction of the hybrid protein M2sHBc, the N–terminus of HBc was used. The HBc sequence contains an arginine–rich C–terminal domain, which binds viral DNA during the viron assembly. When expressed in * E. coli * , ** this domain binds bacterial RNA [23], whose presence in the preparation is undesired. Since the C–terminal domain (150 – 183 amino acid residues) is not necessary for the assembly of the particles [24], it was removed and replaced by a cysteine residue, whose introduction increases the stability of HBc particles [16]. Therefore, our hybrid protein M2sHBc comprises, starting from the N–terminus, the sequence of M2e peptide of the swine flu virus A/California/04/2009 (H1N1), the sequence of HBc antigen from the 4^th^ to 149^th^ amino acid residues, and the C–terminal cysteine.

**Fig. 1:**
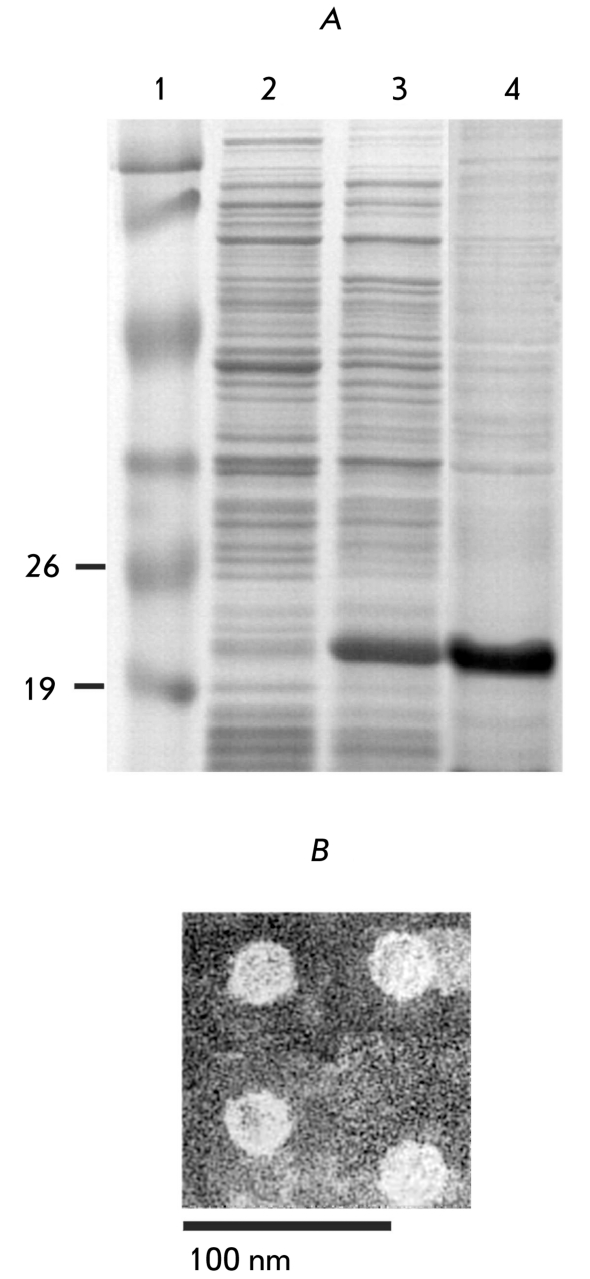
Expression and purification of М2sНВс particles. (А) SDS-PAGE analysis of protein samples. 1 - molecular weight marker, kDa. 2 - protein sample from the strain DLT1270/ pQE- M2sHBc before the induction of М2sНВс expression. 3 - the same as in line 2, but after 16h induction of M2sHBc expression. 4 - purified M2sHBc particles (В) Electron microscopy of М2sНВс particles.

The gene coding for the hybrid protein M2sHBc was synthesized using three–stage PCR with the HBc sequence as a template. During each stage, the sequences encoding the regions of M2e were added to the 5’–end of the synthetic gene. The obtained synthetic gene M2sHBc was cloned in the expression vector pQE60 (Qiagen) under the control of the promoter, inducible by IPTG. The hybrid protein is well expressed in * E. coli * (Fig. 1A) and mainly present in the soluble fraction. The assembly of M2sHBc in virus–like nanoparticles was confirmed by electron microscopy of a purified specimen (Fig. 1B).

**Immunogenicity of the M2sHB particles ** Mice immunization with the purified preparation of M2sHBc particles was done to characterize their immunogenicity and protectivity. The test group that consisted of 60 animals was immunized subcutaneously, using TiterMax Gold Adjuvant (Sigma) for the first vaccine introduction, and the Freund’s adjuvant (Sigma) for the consecutive immunizations. To test the immunogenicity of the candidate vaccine, the mice sera were analyzed two weeks after the first and the third immunizations and antibody titers were determined in the pooled mice sera from each group (3–5 mice). To perform ELISA, we used four synthetic peptides whose sequences corresponded to the M2e of the swine flu virus A/California/04/2009, two strains of avian flu, and a human strain, A/PR/8/34. The results obtained (Table 3) show that after three immunizations the serum antibodies of isotope IgG are produced in high titers. These antibodies bind both synthetic peptides G–26 of the swine flu A/California/04/2009 (H1N1), the sequence of which matched one in M2sHBc used for the immunization, as well as synthetic peptides, the sequences of which correspond to the M2e of heterological strains of the avian and human influenza.

**Table 3:** Titers of IgG antibodies against M2e in sera of immunized mice.

Serum samples	Titers of antibodies recognizing synthetic M2e peptides
G-26	G-19	G-11-1	G-18
After first immunization	1600	1600	800	800
After third immunization	51200	51200	51200	6400
Positive control (monoclonal antibodies against G19 peptide, clone D2)	>51200	>51200	>51200	1600
Negative control (sera of nonvaccinated mice)	<400	<400	<400	<400

**Protective action of the candidate vaccine ** In order to evaluate the effectiveness of the vaccine, mice in both the experimental and control groups were challenged with three influenza strains adapted to mice: A/Duck/Potsdam/1402–6/1986 (H5N2), A/California/04/2009 (H1N1), and A / PR / 8 / 34 (H1N1). Viruses were administered intranasally at a dose of 5 LD50.

**Fig. 2:**
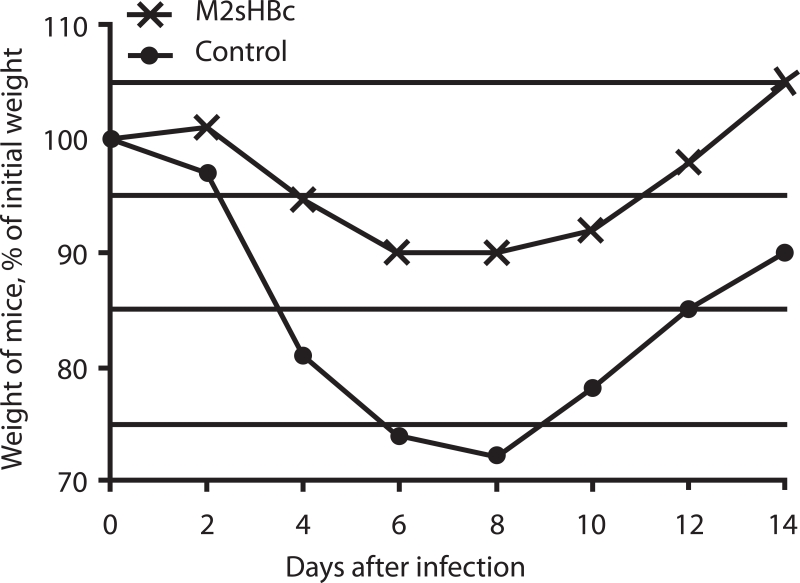
Dynamics of body weight of mice after challenge with influenza virus A/California/04/2009 (H1N1). Data in the control are shown for survived mice.

Fig. 2 shows the body weight loss dynamics of animals after infection with 5 LD50 of the swine flu virus A/California/4/2009, which could indicate the severity of the disease. The weight of the immunized animals dropped after infection (to 90% of the initial weight), but to a much lesser extent than that of mice in the control group (up to 70% of the initial weight). These results indicate that immunization with candidate vaccines will not prevent influenza infection, but that it will reduce the morbidity.

**Fig. 3:**
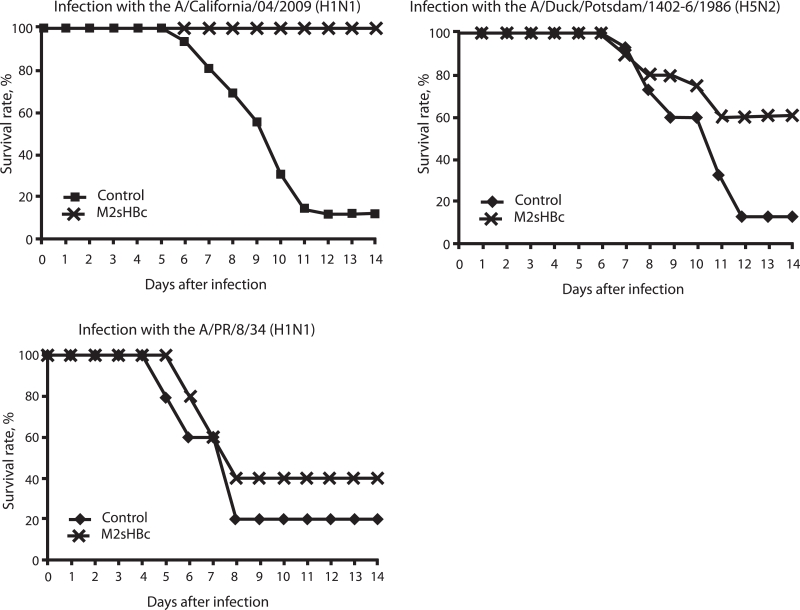
Survival of mice immunized with M2sHBc, followed by a potentially lethal challenge with different mouse-adapted influenza strains.

The dynamics of mice death after infection with various influenza strains are shown in Fig. 3. The results suggest that M2sHBc provides complete protection after a triple immunization. Over the entire period of observation, all of the animals in this group survived, whereas in the control group of mice subjected to these same infection conditions only 12% of animals survived. Partial protection against infection was observed against influenza strains in which the amino acid sequence of the M2e peptide differs from the one used in the preparation for immunization. Thus, 60% of immunized animals survived upon infection with the avian flu A/Duck/Potsdam/1402–6/1986 as opposed to 12% in the control group (statistical significance P < 0.006, Fisher test). When infected with a “human” influenza strain A/PR/8/34, the survival rate of animals was 40% in the experimental group and 20% in the control.

**Prospects for the development of universal influenza vaccines based on M2e**. The conservation of an amino acid sequence of the M2 protein has become a basis for the development of a universal influenza vaccine. Since practically all type A influenza viruses isolated from the human population have the same sequence of M2e, the prospects for the creation of such a vaccine are real [5]. However, strains of animal origin, such as the “swine flu” virus, that have appeared in recent years have differences in the M2e peptide sequence, and, as shown through our results, the effectiveness of an M2e–based vaccine against heterologous strains of influenza is lower. One of the ways to create a M2e vaccine effective against a wide range of influenza strains, both human and animal, can be through the inclusion of several copies of M2e peptide sequences corresponding to different types of M2e into the M2sHBc particles.

## CONCLUSIONS

The purpose of this study was to develop a recombinant candidate vaccine against a new, highly pathogenic strain of the influenza A virus: the swine flu H1N1. We used an approach intended to design a nanovaccine in which an extracellular domain of the M2 protein of the influenza virus was introduced on the surface of the virus–like particles formed by a nuclear antigen of hepatitis B. Our data show that the hybrid protein M2sHBc was efficiently expressed in * E. coli * and self–assembled in nanosized virus–like particles. Mice immunization with M2sHBc particles generates an effective immune response against M2e, and it ensures immunity against an influenza virus strain that has an identical M2e peptide sequence. Thus, M2sHBc particles can be used as a basis for the development of a recombinant vaccine against the modern pandemic swine flu H1N1 and other viruses whose appearance is expected in the coming years.

## REFERENCES

1NicholsonK.WebsterR.HayA.Textbook of InfluenzaBlackwell ScienceOxford19982WebsterR.BeanW.GormanO.ChambersT.KawaokaY.Microbiol.19925615217910.1128/mr.56.1.152-179.1992PMC37285915791083WebbyR.PerezD.ColemanJ.Lancet.2004363109911031506402710.1016/S0140-6736(04)15892-3PMC71124804NeirynckS.DerooT.SaelensX.Nat. Med.19995115711631050281910.1038/134845SchotsaertM.De FiletteM.FiersW.SaelensX.Expert Rev Vaccines.200984995081934856510.1586/erv.09.6PMC27063896LambR.ZebedeeS.RichardsonC.Cell198540627633388223810.1016/0092-8674(85)90211-97PintoH.HolsingerJ.LambA.Cell199269517528137468510.1016/0092-8674(92)90452-i8FiersW.De FiletteM.BirkettA.NeirynckS.Min JouW.Virus Res.20041031731761516350610.1016/j.virusres.2004.02.0309ItoT.GormanO.KawaokaY.BeanW.WebsterR.J. Virol.19916554915498189539710.1128/jvi.65.10.5491-5498.1991PMC24904310FengJ.ZhangM.MozdzanowskaK.J. Virol.2006310210210.1186/1743-422X-3-102PMC17023541715010411De FiletteM.Min JouW.BirkettA.Virology20053371491611591422810.1016/j.virol.2005.04.00412IonescuR.PrzysieckiC.LiangX.J. Pharm. Sci.20069570791631522810.1002/jps.2049313BessaJ.SchmitzN.HintonH.Immunol.20083811412610.1002/eji.2006369591808103714DenisJ.Acosta-RamirezE.ZhaoY.Vaccine200826339534031851115910.1016/j.vaccine.2008.04.05215Meshcheriakova Iu.A.El’darovM.A.MigunovA.I.Mol. Biol.Moscow2009437417501980703816De FiletteM.FiersW.MartensW.Vaccine200624659766011681443010.1016/j.vaccine.2006.05.08217TompkinsS.ZhaoZ.LoC.Emerg. Infect. Dis.2007134264351755209610.3201/eid1303.061125PMC272589918GrantS.JesseeJ.BloomF.HanahanD.Proc. Natl Acad. Sci. USA19908746454649216205110.1073/pnas.87.12.4645PMC5417319WynneS.CrowtherR.LeslieA.Mol. Cell.199937717801039436510.1016/s1097-2765(01)80009-520KratzP.BottcherB.NassalM.Proc. Natl. Acad. USA1999961915192010.1073/pnas.96.5.1915PMC267111005156921MurrayK.ShiauA.L.Biol. Chem.19993802772831022332910.1515/BC.1999.03822PumpensP.GrensE.Intervirology.200144981141150987110.1159/00005003723WingfieldP.StahlS.WilliamsR.StevenA.Biochemistry.19953449194932771101410.1021/bi00015a00324ZhengJ.SchodelF.PetersonD.J. Biol. Chem.1992267942294291577770
